# Primers and copper responsive promoter design and data of real-time RT-PCR assay in filamentous fungus *Trichoderma reesei*

**DOI:** 10.1016/j.dib.2017.11.018

**Published:** 2017-11-07

**Authors:** Wei Wang, Yumeng Chen, Dong-Zhi Wei

**Affiliations:** State Key Lab of Bioreactor Engineering, New World Institute of Biotechnology, East China University of Science and Technology, Shanghai, China

**Keywords:** *Trichoderma reesei*, Filamentous fungus, Copper responsive promoter, Quantitative real-time PCR, Plasmid construction, Gene expression

## Abstract

This data article contains data related to the research article entitled “Copper-mediated on-off control of gene expression in filamentous fungus *Trichoderma reesei*” (Wang et al., 2017) [1]. Four kinds of copper responsive promoters were designed. Quantitative PCR (qPCR) was performed to determine relative mRNA levels of red fluorescent protein gene (*rfp*) extracted from cells grown under different concentrations of CuSO_4_. Three deletion vectors were constructed by using a copper-mediated self-excision cassette instead of a xylose-mediated self-excision cassette (Zhang et al., 2016) [2] to knock out *xyn1*, one of the two major specific endo-β-1,4-xylanases (Rauscher et al., 2006) [3], *xyr1*, the key transcriptional activator of cellulolytic and xylanolytic genes (Lichius et al., 2015) [4], and *ace3*, a factor essential for cellulase production (Häkkinen et al., 2014) [5]. This data article reports the primers, vector construction, and qPCR assay.

**Specifications Table**

**Table:** 

Subject area	*Biology*
More specific subject area	*Molecular biology, vector construction, Quantitative real time PCR*
Type of data	*Table, figure*
How data was acquired	*Sequencing data were acquired through NCBI. In silico analysis of gene using online Real-time PCR (TaqMan) Primer Design (GenScript, China) and primer design software version 6.0 (Premier Biosoft, USA).*
Data format	*Raw, analyzed*
Experimental factors	*Gene sequences were retrieved from GenBank database; Plasmid were constructed; rfp expression were analyzed by qRT-PCR*
Experimental features	*Four kinds of copper responsive promoters were designed. qRT-PCR was performed to determine relative red mRNA levels of rfp extracted from cells grown under different concentrations of CuSO*_*4*_*. Three deletion cassettes were constructed to knockout xyn1, xyr1, and ace3, respectively.*
Data source location	*Shanghai, China*
Data accessibility	*Data is provided with this article*

**Value of the data**

• The modified copper responsive promoter Ptcu1c from *T. reesei* was used for the copper-dependent on-off control of DNA transcription and protein expression.
• The relative levels of *rfp* transcripts increased ~500-fold in the absence or presence of copper.
• The copper-mediated self-excision cassette was more widely used than a xylose-mediated self-excision cassette in some *T. reesei* disruptants for the screening of candidate regulators for cellulase and hemicellulase production.

## 1 Data

Four copper responsive promoters were designed. Quantitative real-time PCR (qRT-PCR) was performed to determine relative mRNA levels of *rfp* extracted from cells grown under different concentrations of CuSO_4_. By using the copper-mediated self-excision cassette, three deletion plasmids were constructed to knockout *xyn1*, *xyr1*, and *ace3*.

## 2 Experimental design, materials and methods

### 2.1 Modified copper responsive promoters

Sequences of native P_*tcu1*_ (1715 bp) of *Trichoderma reesei* were downloaded from the genome sequence of *T. reesei* QM6a (http://genome.jgi-psf.org/Trire2/Trire2.home.html). Three truncated promoter forms, P_*tcu1a*_ (1249 bp), P_*tcu1b*_ (1085 bp), and P_*tcu1c*_ (535 bp), were randomly selected by us. The primers were designed using Primer Premier 6.0. The overlap sequences, “TTAATTAAGTTAACTCTAGA” and “CACGTGATGACCCGACGTC” were added to the 5′ ends of forward and reverse primers, respectively. Four kinds of copper responsive promoters were cloned by primers (Table 1).

**Table 1:** Detailed information on copper responsive promoter primers.

Name	Sequences (5′-3′)	Relevant gene
Pcu1-f	TTAATTAAGTTAACTCTAGAGCGGAATCCTACATTCCCAGAT	Pcu1
Pcu1-r	GACGTCGGGTCATcacgtgGGCCATTGTCGTATCAACCAGGTCGTA	
Pcu1a-f	TTAATTAAGTTAACTCTAGAGCATTACAGACAGAGGCGTGAG	Pcu1a
Pcu1a-r	GACGTCGGGTCATcacgtgGGCCATTGTCGTATCAACCAGGTCGTA	
Pcu1b-f	TTAATTAAGTTAACTCTAGAAGGCTGACTAGAACCACAACTTG	Pcu1b
Pcu1b-r	GACGTCGGGTCATcacgtgGGCCATTGTCGTATCAACCAGGTCGTA	
Pcu1c-f	TTAATTAAGTTAACTCTAGAGCAGCCAGATAAGTTCAATACC	Pcu1c
Pcu1c-r	GACGTCGGGTCATcacgtgGGCCATTGTCGTATCAACCAGGTCGTA	

### 2.2 Expression levels of rfp in T. reesei transformants

About 100 mg of *T. reesei* mycelium was harvested, and grown under different concentrations of CuSO_4_ for 36 h. Total RNA was extracted using a FastRNA Pro Red Kit (MPbio, Irvine, CA, USA), according to the manufacturer's instructions. Reverse transcription was performed with 1000 ng of total RNA, using the TransScript All-in-One First-Strand cDNA Synthesis SuperMix for qPCR (TransGen, Beijing, China), according to the manufacturer's instructions. For RT-qPCR, the TransStart TipTop Green qPCR SuperMix (TransGen) was used with 200 nM of forward and reverse primers (Table 2) and 1 μL of 10-fold diluted cDNA in a final volume of 20 μL. For gene transcription analysis, SYBR green assays, using primers with the reference gene *sar1,* were performed as described in the previous publication [6]. The primers of *rfp* were designed using GenScript Real-time PCR (TaqMan) Primer Design (https://www.genscript.com/tools/real-time-pcr-tagman-primer-design-tool). Thermocycling was performed in an ABI StepOne Plus thermocycler (Applied Biosystems, Foster City, CA, USA).

**Table 2:** Primers used in quantitative real-time PCR (qRT-PCR).

Name	Sequences (5′-3′)	Relevant gene
q-sar1-f	TGGATCGTCAACTGGTTCTACGA	qRT-PCR
q-sar1-r	GCATGTGTAGCAACGTGGTCTTT	
q-rfp-f	GCTTCAAGGTGCGCATGGAG	
q-rfp-r	CGGTGTTGTGGCCCTCGTAG	

**Fig. 1:**
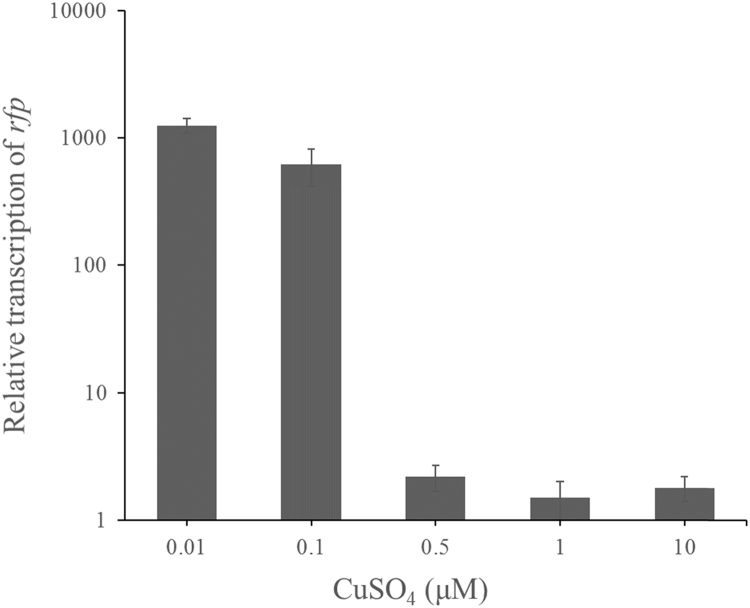
Expression levels of *rfp* in the absence and presence of high levels of copper. The mRNA level of an addition of 0.5 μM copper was set as 2. Error bars indicate mean±SD (*n*=3 samples) from the same experiment.

Quantitative real-time PCR (qRT-PCR) was performed using Ptcu1c-*rfp* [1] to determine relative *rfp* mRNA levels extracted from cells grown under different concentrations of CuSO_4_ (Fig. 1). The relative levels of *rfp* transcripts increased ~500-fold in the absence or presence of high levels of copper, indicating that the on-off control functions by affecting target RNA levels.

### 2.3 Deletion plasmid construction

The 500–1000 bp length of 5′-ends and 3′-ends of the sequences of *xyn1* [3], *xyr1* [4], and *ace3* [5] were PCR-amplified from *T. reesei* Qm9414 or RUT C30 genomic DNA using the appropriate primers (Table 3). The primers were designed using Primer Premier 6.0. The resulting fragments were sequentially fused to the *Pac*I/*Xba*I and *Swa*I sites of LML4.0 [2] using the Seamless Cloning Kit (TransGen Biotech, Beijing, China) to generate the vectors Dxyn1, Dxyr1, and Dace3 (see Fig. 1 in Ref. [1]). All plasmids were confirmed via DNA sequencing.

**Table 3:** Primers used in deletion plasmids construction.

Name	Sequences (5′-3′)	Relevant gene
XYN15-F	GATTACGAATTCTTAATTAACCAGCATCTGTCTAGTTGTGGAGATATG	*xyn1*
XYN15-R	TTAAGTTAACTCTAGACCTTGAAGTCGATACTATGCAGTTGAG	
XYN13-F	ACTAGTGAGCTCATTTGTTCTGTTGATGTTGACTTGGAG	
XYN13-R	AGTGCCAAGCTTATTTGACTGAAGGCGATGTTCTCTG	
XYR15-F	GATTACGAATTCTTAATTAAACGAGTATCTCCGAAATTCCCTTTGG	*xyr1*
XYR15-R	TTAAGTTAACTCTAGAGCGCTGTGTGCGATGTGAAG	
XYR13-F	ACTAGTGAGCTCATTTGGAGGCCACTCAATCGTATGACG	
XYR13-R	AGTGCCAAGCTTATTTGAACCTCTTACTCACATTCACTTGACTTG	
ACE35-F	GATTACGAATTCTTAATTAATCTCTGTTGTCATTGCTCCTCCT	*ace3*
ACE35-R	TTAAGTTAACTCTAGAGGCTGGTCGCTCTTCTTCCT	
ACE33-F	ACTAGTGAGCTCATTTGCCATCATCCATCGCAACCA	
ACE33-R	AGTGCCAAGCTTATTTCCATAGGTAGCCAGTTCGTATCC	
