# Supplementary material for: Primers and copper responsive promoter design and data of real-time RT-PCR assay in filamentous fungus Trichoderma reesei
Source: Data Brief. 2017 Nov 7;16:109–13. doi: 10.1016/j.dib.2017.11.018 (PMC5694953; doi:10.1016/j.dib.2017.11.018)
Supplement: Supplementary file 1 — Supplementary material [file mmc1.docx]

**Competing interests**

The authors (Wei Wang, Yumeng Chen, and Dong-Zhi Wei) declare no conflict of interest.

Wei Wang
