# Supplementary material for: Primers and copper responsive promoter design and data of real-time RT-PCR assay in filamentous fungus Trichoderma reesei
Source: Data Brief. 2017 Nov 7;16:109–13. doi: 10.1016/j.dib.2017.11.018 (PMC5694953; doi:10.1016/j.dib.2017.11.018)
Supplement: Supplementary file 2 — Supplementary material [file mmc2.doc]

Dear editor:

We assure the content of this paper has never been published elsewhere. All authors have contributed significantly to this study and all of them are in agreement with the submission. We declare there is no conflict of interest.

Wei Wang
